# Comparative Analyses of Chromatin Landscape in White Adipose Tissue Suggest Humans May Have Less Beigeing Potential than Other Primates

**DOI:** 10.1093/gbe/evz134

**Published:** 2019-06-24

**Authors:** Devjanee Swain-Lenz, Alejandro Berrio, Alexias Safi, Gregory E Crawford, Gregory A Wray

**Affiliations:** 1 Biology Department, Duke University; 2 Duke Center for Genomic and Computational Biology, Duke University; 3 Division of Medical Genetics, Department of Pediatrics, Duke University

**Keywords:** comparative genomics, chromatin accessibility, primates, adipose

## Abstract

Humans carry a much larger percentage of body fat than other primates. Despite the central role of adipose tissue in metabolism, little is known about the evolution of white adipose tissue in primates. Phenotypic divergence is often caused by genetic divergence in *cis*-regulatory regions. We examined the *cis*-regulatory landscape of fat during human origins by performing comparative analyses of chromatin accessibility in human and chimpanzee adipose tissue using rhesus macaque as an outgroup. We find that many regions that have decreased accessibility in humans are enriched for promoter and enhancer sequences, are depleted for signatures of negative selection, are located near genes involved with lipid metabolism, and contain a short sequence motif involved in the beigeing of fat, the process in which lipid-storing white adipocytes are transdifferentiated into thermogenic beige adipocytes. The collective closing of many putative regulatory regions associated with beigeing of fat suggests a mechanism that increases body fat in humans.

## Introduction

Humans have a remarkable amount of body fat. While other primates have <9% subcutaneous fat in the wild, the derived state in healthy humans is to maintain 14–31% body fat (Zihlman and Bolter 2015; Pontzer et al. 2016). Although little is known about white adipose tissue (WAT) evolution in primates, a growing body of evidence suggests that humans have uniquely adapted WAT to support the high energy needs of our brains (Haygood et al. 2007; Babbitt et al. 2011; Pfefferle et al. 2011; Bozek et al. 2014, 2015; Bauernfeind et al. 2015; Blekhman et al. 2015; Pontzer et al. 2016). To better understand the evolution of increased body fat in humans, a direct comparison between human and nonhuman primate adipose tissue is needed.

Here, we present a comparative analysis of the chromatin landscape in human and chimpanzee WAT. We mapped open chromatin regions (OCRs), which are highly enriched for enhancers, promoters, and other transcriptional regulatory elements. We used rhesus macaque WAT to polarize specific open chromatin changes to either the human or chimpanzee branch. We detected 2,992 regions that are differentially accessible between human and chimpanzee. Notably, we find that OCRs that are less accessible in humans relative to chimpanzee and rhesus macaque are enriched for *cis*-regulatory elements, depleted for signatures of adaptive constraint, and are specifically near genes involved with lipid metabolism. These regions are also enriched for a sequence motif that binds a transcription factor involved in the browning and beigeing of fat, the differentiation of mesenchymal stem cells into brown adipocytes and the transdifferentiation of lipid-storing white adipocytes into thermogenic beige adipocytes, respectively. The data suggest that shutting down beigeing pathways through chromatin regulation drove increased WAT accumulation in humans.

## Results

### Open Chromatin Region Profiles Are Unique to Species

We generated Assay for Transposase-Accessible Chromatin sequencing (ATAC-seq) data on white adipose samples from humans, chimpanzees (*Pan troglodytes*), and rhesus macaque (*Macaca mulatta*) (supplementary table 1, Supplementary Material online) (Buenrostro et al. 2015). We mapped reads from each technical replicate to the sample’s native reference genome assembly. For nonhuman primates, we only retained reads that could be reciprocally converted between the human genome hg19 and the native genome using the genome conversion tool liftOver (Kent et al. 2002). To prevent mapping biases, we performed a reciprocal liftOver from hg19 to panTro4 (chimpanzee) and back to hg19 for human samples. We called OCRs for each biological replicate using MACS2 (Zhang et al. 2008) and generated a union set of OCRs from all three species. OCRs that contained zero reads for any sample, which is an indication of mapping problems, were removed from the analysis. Our final OCR set contained 160,625 OCRs (supplementary table 2, Supplementary Material online). We used adipose ChromHMM predictions to characterize the function of OCRs (supplementary fig. 1 and table 3, Supplementary Material online) (Ernst and Kellis 2012). Eighty-seven percent of OCRs are located >5 kb from the closest transcription start site, which indicates that our ATAC-seq protocol can identify distal regulatory regions in WAT (supplementary fig. 1*A* and *B*, Supplementary Material online).

**Fig. 1.**
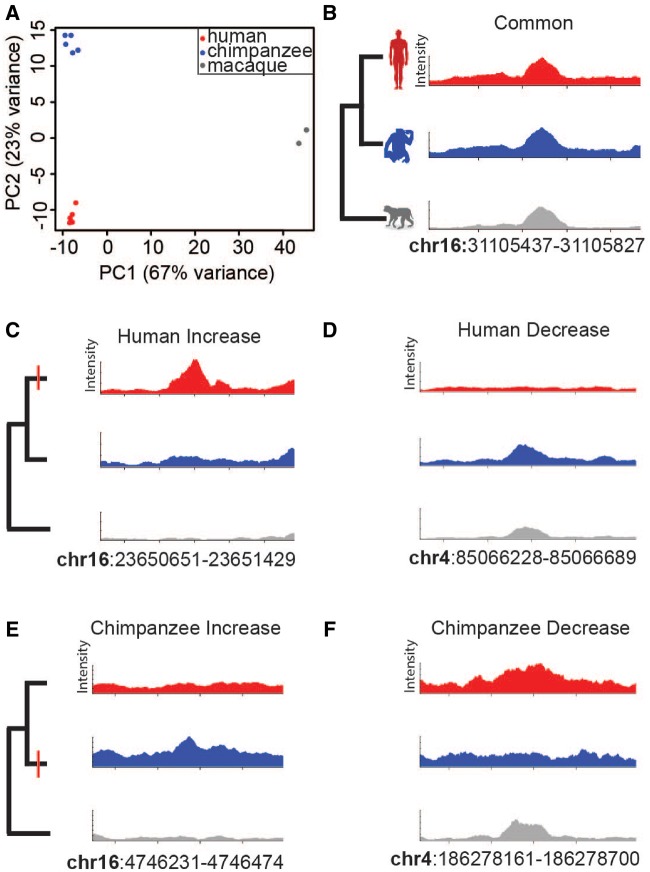
—Detection of species-specific OCR state changes. (*A*) Principal component analysis of OCRs in human, chimpanzee, and rhesus macaque adipose. Note that intraspecific variation is much smaller than interspecific variation. A common OCR state is depicted in (*B*), where the *x*-axis represents chromosome coordinates and the *y*-axis represents OCR intensity from MACS2. Human-specific OCR state changes (red dash) to increased accessibility (*C*) and to decreased accessibility (*D*) from ancestral state (i.e., rhesus macaque accessibility). Chimpanzee-specific OCR state changes (red dash) to increased accessibility (*E*) and to decreased accessibility (*F*) from ancestral state. Genomic coordinates of the OCR of interest are listed.

To understand general patterns of OCRs, we performed principal component analysis (PCA) on normalized count data (fig. 1*B*). The first eigenvector explains 67% of the variance and separates rhesus macaque samples from chimpanzee and human samples. The second eigenvector explains 23% of the variance and separates human and chimpanzee samples. Technical replicates correlate highly (Pearson > 0.85) and are more similar to one another than biological replicates within a species (supplementary fig. 1*C* and *E*, Supplementary Material online). Like most genetically driven phenotypes, OCR profiles reflect the known primate phylogeny, which indicates ATAC-seq data can be used to analyze adipose evolution in primates.

We next used DESeq2 to identify OCR regions that are quantitatively differentially accessible between species. We quantified OCR accessibility rather than simply annotating the presence or absence of an OCR in a species. Since accessibility is a continuous trait, setting an appropriate threshold for presence or absence of a OCR can be difficult. Rather than set an arbitrary threshold, our approach exploits the dynamic range of the continuous data to increase the number of species-specific OCRs observed and increase the power for downstream analyses (supplementary fig. 1*D*, Supplementary Material online).

Using rhesus macaque as an outgroup to assign OCR state changes to either the human or chimpanzee branch (Love et al. 2014), we defined four groups of species-specific state changes (fig. 1 and table 1). Human-increased states (*n* = 732) are OCRs that display similar accessibility between the chimpanzee and the inferred ancestral state (i.e., rhesus macaque) but show increased accessibility specifically on the human branch. Human-decreased states (*n* = 782) consist of OCRs that display similar accessibility between chimpanzee and rhesus macaque but decreased accessibility specifically on the human branch. Chimpanzee-increased (*n* = 1,012) or decreased (*n* = 466) state changes are analogous to those in humans. Species-specific OCRs are increased or decreased by at least 2-fold in comparison to OCRs that are not classified as different between the three species. The number of species-specific OCRs are comparable to species-specific accessibility and *cis*-regulatory regions found in other human comparative studies (Shibata et al. 2012; Villar et al. 2015).

**Table 1:** OCR Groups

OCRs	*N*
Total	160,625
Common matched	3,194
Human-increased	732
Human-decreased	782
Chimpanzee-increased	1,012
Chimpanzee-decreased	466

Our analysis resulted in 98% of OCRs as being classified as similar between humans, chimpanzees, and rhesus macaque (i.e., 98% of OCRs are equally accessible in the three primate species). The complete group of “Common” OCRs displayed a wide range of accessibility intensities that included but are broader than the intensities of species-specific OCRs. To ensure our downstream analyses used a control group that mirrored the intensity of the species-specific OCRs, we created a subset of matched Common OCRs that had ATAC-seq read counts between the 20th and 80th percentiles of the species-specific ATAC-seq read counts (supplementary fig. 1*F*, Supplementary Material online) (Shibata et al. 2012).

We compared the results of our analysis using continuous OCR data to analysis using a binary model to call presence or absence of OCRs with MACS2. The majority of Human-increased OCRs are not identified in other species’ genomes and are considered complete gains (89.5%). Over half of the Human-decreased OCRs are not identified as human OCRs (55.1%) and are considered complete losses. The rest of OCRs with decreased accessibility in humans are identified as human OCRs and are considered partial losses. Similarly, the majority of OCRs with increased accessibility in chimpanzees are not identified in other species and are complete gains (77.2%). The majority of OCRs with decreased accessibility in chimpanzees are not identified as chimpanzee OCRs (95.5%).

To ascertain any differences between partial and complete losses in humans, we analyzed the height features of humans and chimpanzees for these groups. The distributions of complete and partial log2-fold changes overlap. The average log2-fold change between humans and chimpanzees in complete losses is greater in magnitude than in partial losses (supplementary fig. 2*A*, Supplementary Material online). While the log2-fold change is greater in magnitude in complete losses, the absolute change in peak height between chimpanzees and humans is greater in partial losses (supplementary fig. 2*B* and *C*, Supplementary Material online). This may be because the average height of partial losses is higher than those of complete losses regardless of species. As it is difficult to determine whether log2-fold change or absolute change is more biologically relevant, we use both complete and partial losses in the Human-decreased analyses.

### Species-Specific OCR States Correlate with *Cis*-Regulatory Divergence

**Fig. 2.**
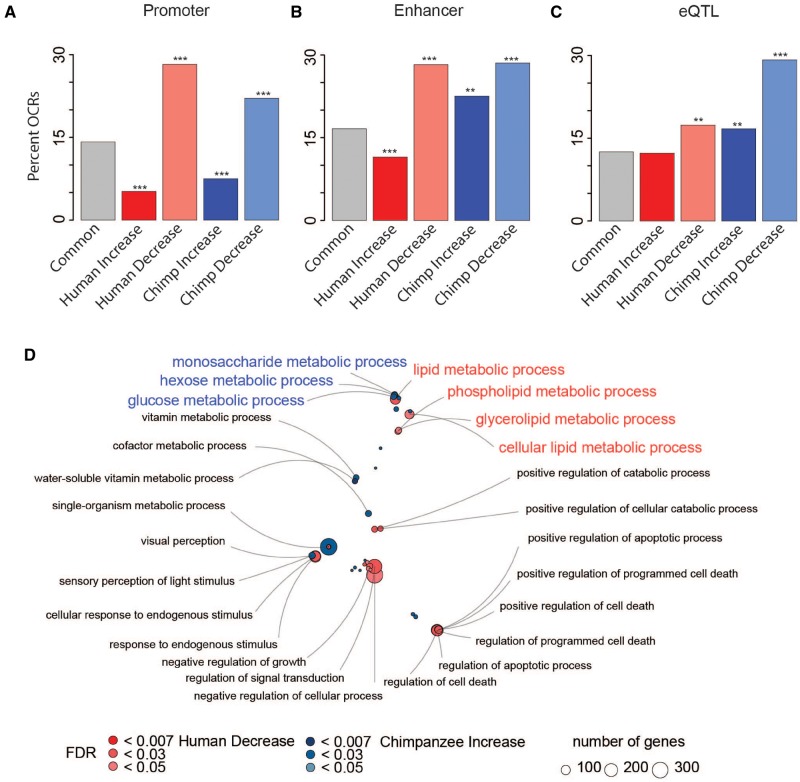
—Species-specific OCR groups are enriched for *cis*-regulatory functions. Species-specific OCR groups enrichment (Fisher’s exact, **P < 0.01, ***P < 0.001) for promoters (*A*) enhancers (*B*), and adipose eQTLs (*C*). GREAT enrichment bubble plot (*D*) with labeled GO terms for bubbles containing at least 25 genes.

To understand the relationship between OCR state and *cis*-regulation, we assigned putative function to each OCR using publicly available human adipose ChromHMM predictions (supplementary table 3, Supplementary Material online) (Ernst and Kellis 2012). Approximately 14% of Common OCRs are predicted to be promoters (fig. 2*A*). OCRs classified as Human-decreased or Chimpanzee-decreased are highly enriched for promoter regions (28.3% and 22.1%, respectively, Fisher’s exact test, *P* < 0.001). In contrast, OCRs classified as a Human-increased or Chimpanzee-increased are significantly depleted for promoters (5.2% and 7.5%, respectively, Fisher’s exact test, *P* < 0.001).

We next compared enhancer ChIP-seq predictions among OCR groups (fig. 2*B*). About 17% of Common OCRs are predicted to be enhancers. Similar to promoters, Human-decreased and Chimpanzee-decreased OCRs are highly enriched for enhancers (28.3% and 28.5%, respectively, Fisher’s exact test, FDR < 0.001). In addition, similar to promoters, Human-increased and Chimpanzee-increased OCRs are not as highly enriched for enhancers. However, Chimpanzee-increased OCRs displayed a higher overlap with enhancers (22.5%, Fisher’s exact test, *P* = 0.008) compared with Human-increased OCRs (11.5%, Fisher’s exact test, *P* < 0.001).

These observations of promoter and enhancer enrichment and depletion reflect expected differences in the pleiotropic effects of OCR state changes in *cis*-regulatory elements. Promoters are necessary and sufficient for basal gene expression, and while enhancers can be necessary for higher expression of some genes, they are not required for low levels of expression. Furthermore, promoters tend to be pleiotropic and function in many cell types, while enhancers are mostly cell type-specific (Consortium et al. 2017). Finally, sequence and function are more conserved in promoters than in enhancers (Villar et al. 2015). The hierarchical importance, pleiotropy, and conservation of promoters compared with enhancers implies that gaining accessibility in promoters is less likely than in enhancers. We observed the number of cell types and tissues in which an OCR is open and found that species-decrease OCRs are open in more cell types and tissues and therefore more pleiotropic than Common OCRs (supplementary fig. 3, Supplementary Material online, Fisher’s exact test, *P* ≪ 0.001). As expected, Human-decreased and Chimpanzee-decreased state changes are more likely to be annotated as promoters than species-increased groups, as well as being significantly more pleiotropic.

The enrichment of species-specific OCR states for *cis*-regulatory regions suggests that species-specific OCR state may be associated with functional expression changes. An association with species-specific OCRs and expression changes would support that the state changes are biologically relevant. To measure association with expression changes, we compared our species-specific OCRs to known human adipose expression quantitative trait loci (eQTL) (Brown et al. 2017).

To determine whether expression changes were enriched in species-specific OCRs, we mapped eQTLs to OCRs (fig. 2*C* and supplementary table 5, Supplementary Material online) (Brown et al. 2017). Interestingly, Human-decreased and Chimpanzee-increased and decrease OCRs are enriched for adipose eQTLs in comparison to Common OCRs (fig. 2*A*, Fisher’s exact test, FDR = change). Human-increased OCRs are not enriched for adipose eQTLs. eQTLs have thus far only been identified in humans, and so we cannot determine whether the same eQTL exists in the chimpanzee population. We asked whether the increase in Human-decreased OCRs could be due to changes in minor allele frequency or differences in the ancestral allele being present in the human population (supplementary fig. 4*A* and *B*, Supplementary Material online). The minor allele frequency is the same across Common and species-specific OCR groups and the ancestral allele is present in the majority of OCRs, indicating recent population dynamics are not influencing groups differently. Therefore, observed species-specific OCR groups are a reflection of divergence between humans and chimpanzees. We also note that eQTL cover a broader range than an OCR, and the “openness” of OCRs is on a continuum. Therefore, an OCR can be labeled as “decreased” while still containing functional eQTLs.

Since species-specific OCRs are enriched for eQTLs, we posited that they could also be enriched for differential gene expression between human and chimpanzee. To test this, we assigned each OCR to the closest transcription start site and compared with published RNA-seq data of WAT from human and chimpanzee (supplementary fig. 4*C* and table 4, Supplementary Material online) (Babbitt et al. 2017). About 5% of Common OCRs are near genes associated with differential gene expression between humans and chimpanzees. Although species-specific OCRs are associated with higher levels of differential gene expression, this increase is not statistically significant.

We next asked whether species-specific OCR states were associated with biological functions. We used GREAT to perform gene ontology enrichment analyses for each OCR category, using the full set of OCRs as a background set (supplementary tables 5–8, Supplementary Material online) (McLean et al. 2010). Similar to the eQTL analyses, Human-decreased and Chimpanzee-increased OCRs are enriched for adipose-relevant gene ontology functions. In particular, they reflect the different diets of the two species: Human-decreased OCRs are located near genes associated with lipid metabolism, while Chimpanzee-increased OCRs are located near genes associated with simple sugar metabolism (fig. 2*D* and supplementary tables 6 and 7, Supplementary Material online). We asked whether completely or partially closed OCRs were driving the signal in Human-decreased OCRs. When we split the data into two groups, there is not enough power to observe enrichment in any biological process. However, this does suggest that completely and partially closed OCRs share similar biological processes.

**Fig. 3.**
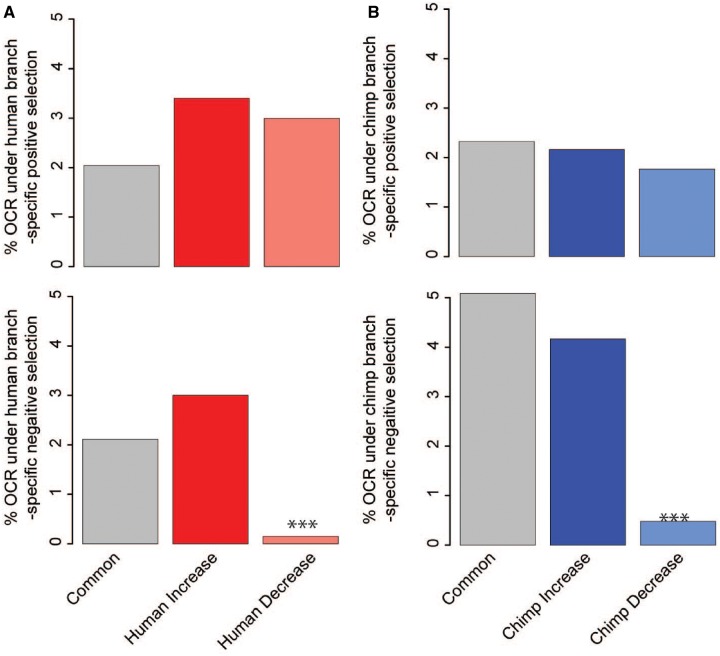
—Branch-specific positive selection as detected by phyloP. Percentage of OCRs under (*A*) human and (*B*) chimpanzee branch-specific positive selection (top panels). Percentage of OCRs under (*A*) human and (*B*) chimpanzee branch-specific negative selection (bottom panels, Fisher’s exact test, ***P < 0.001).

To further explore potential biological functions of species-specific OCR states, we identified regions under positive selection. We utilized two branch-specific tests of positive selection, phyloP and a framework we developed (see Materials and Methods and supplementary tables 9–12 and fig. 5*A* and *B*, Supplementary Material online) (Pond et al. 2005; Haygood et al. 2007). Both methods compute *P* values of acceleration for a given OCR alignment (21–22). We see a high correlation between the *P* values from phyloP and our method (*R* = 0.78 for humans and *R* = 0.80 for chimpanzees). Additionally, there is a large overlap of regions under positive selection between the two methods (supplementary fig. 5*C* and *D*, Supplementary Material online). We compared human-branch specific positive selection in human-specific OCRs to that in Common OCRs and chimpanzee-branch specific positive selection for chimpanzee-specific OCRs to that in Common OCRs. Species-specific OCRs are not enriched for more positive selection in comparison to Common OCRs (2–4% of OCRs using phyloP, fig. 3*A* and *B*—top panels; 3–4% of OCRs using our tool, supplementary fig. 5*E* and *F*, Supplementary Material online).

Many species-specific OCRs under positive selection, regardless of species or state-change, are closest to genes involved with biologically plausible functions for adipose tissue, including browning of fat, cell differentiation, leptin regulation, and obesity and related diseases (supplementary tables 9–12, Supplementary Material online). Although each OCR group has equal amounts of human and chimpanzee branch-specific positive selection, there is little overlap in the genes that are evolving under positive selection on the human and chimpanzee branches. This indicates that humans and chimpanzees are possibly undergoing positive selection for different phenotypes.

We next tested whether species-specific OCRs are under negative selection using the phyloP to compute *P* values for conservation for OCR alignments (fig. 3, bottom panels, see Materials and Methods, and supplementary tables 13 and 14, Supplementary Material online). We compared human-branch specific negative selection in human-specific OCRs to that in Common OCRs and chimpanzee-branch specific negative selection for chimpanzee-specific OCRs to that in Common OCRs (fig. 3*A* and *B*, bottom panels). While Common OCRs and species-specific increased OCRs have a similar percentage of regions under constraint, species-specific decreased OCRs are significantly depleted for regions under constraint.

### Transcription Factor Binding Motifs Characterize Species-Specific OCR States as Being Related to Brown Adipogenesis

Finally, we characterized categories of species-specific OCRs for enrichment of DNA sequence motifs. To control for local sequence features and shared genes, we created a nearest null set of common OCRs for each species-specific OCR state (i.e., for each species-specific OCR, the closest common OCR was used for a null comparison, supplementary fig. 6, Supplementary Material online). To ensure the matched null set was representative of the rest of the genome, we used a machine learning algorithm in the R package gkm-SVM to test whether k-mers could predict the closest common OCRs as from the rest of the genome (Ghandi et al. 2014). We measured the average performance of gkm-SVM to classify a positive set of matched null OCRs from ∼1,100 random sequences from random genomic OCRs from the background (not including any matched null sequences). The matched null sets are indistinguishable from the rest of the genome, which indicates that the matched null sets are good proxies for genomic background sets (supplementary fig. 7, Supplementary Material online).

**Fig. 4.**
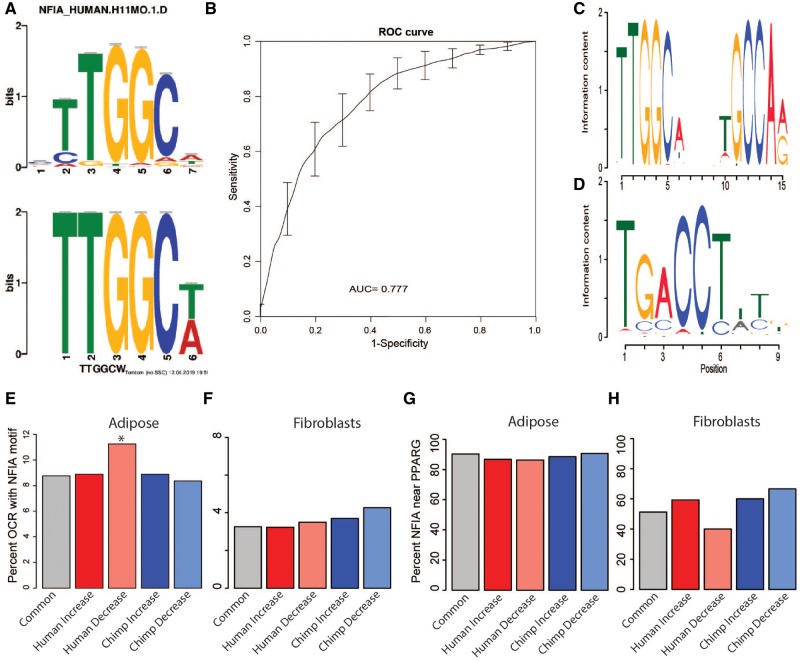
—Human-decreased OCRs are associated with NFIA. We used MEME-ChIP to identify motifs that are enriched in human-decreased OCRS (*A*) and gkm-SVM to distinguish species-specific OCRs from null common OCRS. Shown are the receiver/operating curve for human-decrease (*B*). We scanned OCRs for expanded NFIA (*C*) and PPARG (*D*) motifs. We compared NFIA motifs in adipose (*E*) and fibroblasts (*F*) OCRs and PPARG motifs in adipose (*G*), and fibroblasts (*H*).

To identify motif enrichment between species-specific OCRs and their matched null set, we used MEME-ChIP (fig. 4*A* and supplementary tables 15–18, Supplementary Material online) (Machanick and Bailey 2011). MEME-ChIP identifies the NFIA motif as enriched in Human-Decreased OCRs (fig. 4*A* and supplementary table 16, Supplementary Material online, *P* ≪ 0.001). These results are intriguing since NFIA and the master adipogenesis transcription factor PPARG colocalize to regulate adipogenesis in brown adipocytes as well as in white adipocytes transdifferentiating into beige adipocytes (Hiraike et al. 2017; Pradhan et al. 2017). MEME-ChIP also identifies complete losses as the driver of the NFIA signal in human-decrease regions (i.e., complete losses are enriched for NFIA [*P* ≪ 0.001] while partial losses are not). This indicates that although partial and complete losses share some similarities as seen by the GREAT analysis, there are sequence differences that differentiate the groups. MEME-ChIP also identifies short nucleotide motifs in the other species-specific groups, although they are different motifs than the gkm-SVM, shorter than 6 bp, or have no known function in adipose.

In parallel to MEME-ChIP, we compared each species-specific category to its closest null set again using gkm-SVM. We measured the weights of nonredundant 6-mers and find that each species-specific group is distinguishable from its closest null (fig. 4*B* and supplementary fig. 8 and tables 19–22, Supplementary Material online). Interestingly, we find a small set of 6-mers with higher weights that classify human-decrease OCRs, which correspond to NFIA binding motifs (supplementary table 20, Supplementary Material online). Since colocalization of NFIA and PPARG motifs is correlated with an increase in brown adipocyte gene expression, we could measure how often NFIA and PPARG binding motifs occur in the same OCR.

To confirm that human-decrease and chimpanzee-increase OCR sequences are enriched for NFI motifs, we expanded the 6-mer motifs to the full NFIA motif and scanned all sequences for the NFIA motif (fig. 4*C*) (Weirauch et al. 2014). Human-decreased OCR has a significantly higher percentage of OCRs (11.2%) with the longer NFIA motif than Common OCRs (8.8%, Fisher’s exact test, *P* = 0.038). To further investigate whether the NFIA motif could have function in adipose tissue, we took advantage of NFIA’s colocalization with PPARG, the master regulator of adipogenesis.

We scanned sequences for a PPARG motif (fig. 4*D*) and found that over 80% of NFIA motifs occur with a PPARG motif (Weirauch et al. 2014). Because the PPARG motif is abundant across the genome, we wanted to ensure these observations are not an artifact and are specific to adipose OCRs. We therefore performed the same scans NFIA and PPARG motifs in Common and species-specific OCRs identified in a previous study in fibroblasts, which is the only study to our knowledge to also compare primate OCRs (Shibata et al. 2012). We find that fibroblast OCRs have half the amount of NFIA motifs present in adipose OCRs (fig. 4*E* and *F*). Additionally, only half of the fibroblast OCRs that contain NFIA motifs also contain PPARG motifs (fig. 4*G* and *H*). These findings suggest that co-occurring NFIA and PPARG motifs reflect differences in biological function specific to adipose OCRs.

We asked whether the underlying sequence of NFIA and PPARG motifs have changed between humans and chimpanzees. We scanned the human and chimpanzee orthologous sequence of each OCR for NFIA and PPARG motifs (fig. 4*B* and *C*). Each observed motif received a relative score scaled from 0 (no motif) to 1 (perfect motif). We calculated the sum of relative scores for each orthologous OCR and compared the scores of the motifs between orthologous OCRs. In short, we find OCRs where scores of motifs vary but find no patterns in species-specific OCR groups compared with the Common OCRs. The NFIA and PPARG motifs are not changing faster or slower than expected in the species-specific OCRs, which suggests that either epigenetic factors, *cis*-regulatory changes outside the OCR, transfactors, or a mixture of all three control OCR differentiation between species (supplementary fig. 9 and table 23, Supplementary Material online).

## Discussion

### Divergent Evolutionary Patterns in Human and Chimpanzee Adipose Tissue

To better understand the evolution of increased body fat in humans, we performed comparative analyses on the adipose chromatin landscape in humans, chimpanzees, and rhesus macaques. Interestingly, there seem to be two modes of change in the regulatory landscape within human and chimpanzee adipose tissue. In general, Human-decreased OCRs are enriched for promoters and enhancers compared with Human-increased OCRs (figs. 2 and 3). Human-decreased OCRs are also more enriched for adipose eQTLs, relevant gene ontology, and NFIA motifs related to adipogenesis and beigeing of fat (figs. 2–4). We also find that Chimpanzee-increased OCRs are more closely associated with functional enrichment of promoters, enhancers, relevant gene ontology, and NFIA motifs than Chimpanzee-decreased OCRs (figs. 2*D* and *E* and 3). Many Human-decreased OCRs are located near genes associated with lipid metabolism while Chimpanzee-decreased OCRs are located near genes associated with simple sugar metabolism. These differences in gene ontology association may reflect the differences in the adapted diets of these two species. Taken together, these results suggest that humans shut down regions of the genome to accommodate a high fat diet while chimpanzees open regions of the genome to accommodate a high sugar diet.

### Humans May Have Lower Beigeing Potential than Chimpanzees

Our results further suggest a mechanism that may have contributed to the evolution of increased WAT in humans. The body contains two kinds of adipose tissue. The vast majority is WAT, which is composed primarily of white adipocytes and acts as an endocrine and lipid storage organ. In addition, the body contains brown adipose tissue (BAT), which is comprised primarily of brown adipocytes and whose main role is thermoregulation. Brown and white adipocytes differentiate from distinct mesenchymal cell lineages (Wu et al. 2012; Sepa-Kishi and Ceddia 2018). White adipocytes derive from preadipocyte precursors while brown adipocytes derive from myoblasts, which can also differentiate into muscle cells. Furthermore, brown adipocytes are characterized by many small lipid droplets and a large number of mitochondria, while white adipocytes contain one large lipid droplet and fewer mitochondria.

While WAT derives from a distinct cell lineage and is predominantly made up of white adipocytes, it also contains brown-like cells, called beige or brite adipocytes (Wu et al. 2012; Vargas-Castillo et al. 2017; Hildebrand et al. 2018; Kuda et al. 2018; Sepa-Kishi and Ceddia 2018). Beige adipocytes are a distinct thermogenic fat cell type from brown adipocytes; they derive from the same lineage as white adipocytes and form sporadic pockets within WAT (Wu et al. 2012; Vargas-Castillo et al. 2017; Hildebrand et al. 2018; Kuda et al. 2018; Sepa-Kishi and Ceddia 2018). Beige adipogenesis is induced under a variety of conditions such as cold, caloric restriction, and exercise (Wu et al. 2012; Hildebrand et al. 2018; Sepa-Kishi and Ceddia 2018). Although beige adipocytes stem from the same lineage as white adipocytes, beige cells share characteristics of classical brown fat, such as higher numbers of mitochondria and smaller, more numerous lipid droplets (Wu et al. 2012). Likewise, the transcriptional profile during beige adipogenesis is unique although it shares characteristics with both white and brown adipogenesis (Wu et al. 2012).

In principle, increased WAT in humans could have evolved by shifting differentiation pathways towards white rather than beige adipocytes. Although histology on frozen adipose samples is challenging and do not have a percentage of beige adipocytes in primate fat, we can still observe evidence of browning from the chromatin landscape. The NFIA motif has been implicated in adipogenesis and differences between BAT and WAT (Hiraike et al. 2017; Pradhan et al. 2017). A recent systems biology comparison of murine brown and white adipose found that OCRs enriched in brown adipose contain the NFIA motif and a high enrichment for GO terms involved with browning of fat (Hiraike et al. 2017).

Consistent with these findings, we find the NFIA motif enriched in regions that are specifically closed in human WAT and open in chimpanzee WAT. Human and chimpanzee expression of *NFIA* is similar, and the NFIA motif in the observed OCRs is conserved between humans, chimpanzees, and rhesus macaque. The underlying NFIA motif sequence is not evolving slower or faster than expected in species-specific OCRs (supplementary fig. 9, Supplementary Material online), and decreased OCRs are not under the same level of constraint as Common and increased OCRs (fig. 3). Interestingly, collectively closing *cis*-regulatory regions could be a response to divergence in the diets of humans and chimpanzees, as suggested by the GREAT analyses. Human-decreased regions may be released from the constraints on the NFIA motif due to adaptive dietary changes.

## Conclusions

The data presented here point to a specific molecular mechanism in beige adipogenesis that may have contributed to the derived state of high body fat mass in humans relative to other primates. The ancestral state in nonhuman primates could be maintained by directing white adipose to produce more beige adipocytes. Selective pressure in humans to increase lipid storage for our metabolically demanding brains (Haygood et al. 2007; Babbitt et al. 2011; Pfefferle et al. 2011; Bozek et al. 2014, 2015; Bauernfeind et al. 2015; Blekhman et al. 2015; Pontzer et al. 2016) may have shaped the regulatory landscape to shut down beige pathways and redirect more adipose precursor cells towards white adipocyte identity. The extent to which diet and genetics play a role in accumulating white versus beige adipocytes among primate species remains unexplored. The availability of primate induced pluripotent stem cells means that future studies can begin to disentangle the effects of environment and genetic divergence during adipogenesis (Gallego Romero et al. 2015).

## Materials and Methods

### Tissue Samples and ATAC-Seq

The adipose tissue samples used in this study are listed in supplementary table 1, Supplementary Material online. We obtained reproducible data from three human biological replicates (one to three technical replicates each), two chimpanzee (*Pan troglodytes*) biological replicates (two to three technical replicates each), and one rhesus macaque (*Macaca mulatta*, two technical replicates). Samples were dissected from deceased individuals and sent to us as frozen samples (Babbitt et al. 2017). The low number of biological replicates reflects the difficulty of obtaining nonhuman primate tissue samples.

We homogenized 20 mg of frozen pulverized adipose tissue in nuclei isolation buffer (20 nM Tris–HCl, 50 mM EDTA, 5 mM spermidine, 0.15 mM spermine, 0.1% beta meracptoethanol, 40% glycerol, 1% NP40, pH 7.5) with a dounce homogenizer. The homogenate was centrifuged at 1,100 g for 10 min at 4 °C and the pellets resuspended in resuspension buffer (10 mM Tris–HCl, 10 mM NaCl, 3 mM MgCl_2_, pH 7.4). We ran tagmentation reactions at 37 °C for 30 min, purified samples with Qiagen MinElute kits, and amplified libraries with NEB NextPCR. Duke University’s Sequencing and Genomic Technologies sequenced the libraries with the Illumina 4000 producing 150 bp paired-end reads (supplementary table 1, Supplementary Material online).

### Data Processing, OCR Calling, and Quality Control

We used bowtie2 (Langmead and Salzberg 2012) to map reads from each technical replicate to the sample’s native genome (panTro4 for chimpanzee, hg19 for humans, and rheMac2 for rhesus macaque). For chimpanzee and rhesus macaque samples, we used reciprocal liftOver with human genome hg19 to identify homologous regions between species (Kent et al. 2002). To control for mapping biases due to disparity in genome quality, we used reciprocal liftOver with panTro4 for humans. In other words, we mapped human reads to hg19, used liftOver to convert reads to the panTro4 genome, and used liftOver again to reciprocally convert reads back to the hg19 genome. Unless stated elsewhere, we used hg19 coordinates to analyze the homologous regions.

For each species, we pooled mapped reads from all technical replicates, and used MACS2 to identify OCRs (Zhang et al. 2008). We specified a shift of 100 bp and an extension of 200 bp with an FDR of 0.01. We compiled OCRs from all biological samples and removed any OCR that had 0 read counts from any technical replicate, yielding a final set of 160,625 OCRs with confident 1:1:1 homology among the three species.

### Quantitative Analyses of Differential OCR State

To increase the number of observed state changes in OCRs, we quantified the OCRs based on count data rather than presence or absence of a OCR. We did not use a fold-change threshold to filter out OCRs, because chromosome accessibility is a continuum and setting a threshold can be arbitrary. Additionally, noisy OCRs would drop out of our differential analyses either because one or more technical replicates had 0 read counts or because a differential OCR signal would not be larger than surrounding noise.

DESeq2 (Love et al. 2014) was used to normalize the count data and calculated the Pearson correlation between technical replicates. We retained replicates that correlated well with other technical or biological replicates (*R* > 0.85) for our differential analyses. To determine whether species had an effect on OCRs accessibility, we compared a linear model with a species component (OCR ∼ species) to a null model (OCR ∼ 1) in DESeq2. We assumed the known species tree and used pairwise contrasts between species and rhesus macaque as an outgroup to determine derived OCR state changes in human and chimpanzee (FDR < 0.05). OCRs without a significant species effect (FDR > 0.05) were labeled as Common OCRs. Additionally, we required that the magnitude of difference between humans and chimpanzees be at least a 2-fold difference. Furthermore, we wanted to ensure that the set of common OCRs were similar in read intensities and size as species-specific OCRs. Therefore, we created a matched common set of OCRs that fell in 20–80th percentile of species-specific normalized read count and size.

DESeq was developed for RNA-seq data and ATAC-seq data is inherently noisier than RNA-seq data. Therefore, we would expect a high false-negative rate, which may affect derived OCR groups differently. Short of spiking in true positive reads, which are thus far unverified, we cannot reliably calculate the false negative rate. We did take advantage of the false discovery rate, to plot the percentage of OCRs called as derived state changes as the false discovery rate increases (supplementary fig. 1D, Supplementary Material online). We would expect that the lower the false discovery rate, the number of false negatives being called as positives would grow. Decreased OCR groups may be more difficult to identify as a true positive than Increased OCR groups. This may be due to the lack of power to identify decreased OCRs. Further we analyzed the distribution of derived OCR states across the genome. Based on enrichment tests, no chromosome is depauperate for any derived OCR state (*P* > 0.1). However, we may not have the power to detect depletion of OCR states across any given region due to the low number of derived OCR states.

### Gene Expression Analyses

To gain insight into *cis*-regulatory function of species-specific OCR state, we measured enrichment of OCR with eQTL and chromatin annotations (Ernst and Kellis 2012). We used GREATversion 3.0.0. (http://great.stanford.edu/public/html/, last accessed April 9, 2019) (McLean et al. 2010) to determine whether sets of OCRs possibly regulate genes that are enriched in a biological process. We used species-specific OCR states as our test regions, and the full set of OCRs for our background regions.

To associate differential gene expression with OCR state, we reanalyzed data from Babbitt et al. (2017). We filtered out genes with 0 reads from any biological replicate and used DESeq2 to compare a linear model with a species component (expression ∼ species) to a null model (expression ∼ 1). We assigned enhancers to their closest transcription start site to subset the gene expression data for each OCR group, and used Wilcoxon tests to measure differences in gene expression between OCR states.

### Selection Analyses

We used the SPH method in phyloP to test for branch-specific positive and negative selection for each OCR alignment (Siepel et al. 2006). We computed *P* values for either positive or negative selection of species-specific OCR states in respect to a neutral model of evolution for set of genomic regions that are predicted to be nonfunctional based on functional annotations (Ernst and Kellis 2012).

We also used framework developed by Haygood et al. (2007) to test for branch-specific positive selection. This framework measures the likelihood ratio of an alternative model under positive selection relative to a null model of divergence due to drift and negative selection. This test produces a *P* value associated to ζ, that is analogous to ω, in which ζ < 1 is indicative of a region under negative selection; ζ = 1 is indicative of region under neutral evolution; and ζ > 1 is indicative of a region of positive selection. We compared selection of species-specific OCR states to the same set of genomic regions that are predicted to be nonfunctional as in the phyloP analyses (Ernst and Kellis 2012).

### Motif Analyses

To determine if OCR sequences could be differentiated from the rest of the genome, we used the default settings of the machine learning R package, gkm-SVM (Ghandi et al. 2014). We calculated the average performance of 100 iterations for each OCR set, using a negative group of 1,100 random sequences from the total OCR set. We used the default settings of gkm-SVM to predict species-specific OCR sequences from matched null OCR sequences, which consisted of the closest common OCR to a species-specific null. The match null set controls for shared genes and local genomic features such as GC content (supplementary fig. 5, Supplementary Material online).

To complement the gkm-SVM analyses, we used MEME-ChIP’s differential motif enrichment pipeline from MEME Suite (Machanick and Bailey 2011). http://meme-suite.org/, last accessed April 7, 2019. We used a first-order model to adjust for dimer biases and allowed DREME and MEME to search up to ten motifs. We used CentriMo to search in local mode to find uncentered motifs.

We used TOMTOM from MEME Suite (Gupta et al. 2007) to identify transcription factor candidates that bind to predicted motifs from gkm-SVM and MEME-ChIP. We allowed incomplete alignment, and the Pearson correlation coefficient was calculated for human motifs in HOCOMOCO database (v11 CORE). *P* values were corrected by a Bonferroni correction. We used the R package JASPAR TFBSTools (Tan and Lenhard 2016) to scan sequences for the NFIA (M3607_1.02) and PPARG (M6434_1.02) motifs from CIS-BP Database (supplementary table 11, Supplementary Material online) (Weirauch et al. 2014) . http://cisbp.ccbr.utoronto.ca/, last accessed August 17, 2018. 

## Supplementary Material

Supplementary data are available at *Genome Biology and Evolution* online.

## Supplementary Material

Supplementary_Matrial_evz134

## Notes

**Data deposition:** Raw fastq files can be found at Gene Expression Omnibus (GEO) under accession numbers GSM3494237–GSM3494249. All processed data can be found in the supplementary tables, Supplementary Material online. Scripts for our positive selection framework are available at https://github.com/wodanaz/adaptiPhy. The remaining scripts can be found at https://github.com/devjanee/adipose_atac_seq.
