# Supplementary material for: Comparative Analyses of Chromatin Landscape in White Adipose Tissue Suggest Humans May Have Less Beigeing Potential than Other Primates
Source: Genome Biol Evol. 2019 Jun 24;11(7):1997–2008. doi: 10.1093/gbe/evz134 (PMC6648876; doi:10.1093/gbe/evz134)
Supplement: Supplementary_Matrial_evz134 [file supplementary_matrial_evz134.zip › Supplemental_Figure_FINAL.pdf.tar.gz!/Users/ds394/Documents/ATAC_seq/ATAC_manuscript/GBE_review_files/Supplemental_Figure_FINAL.pdf]

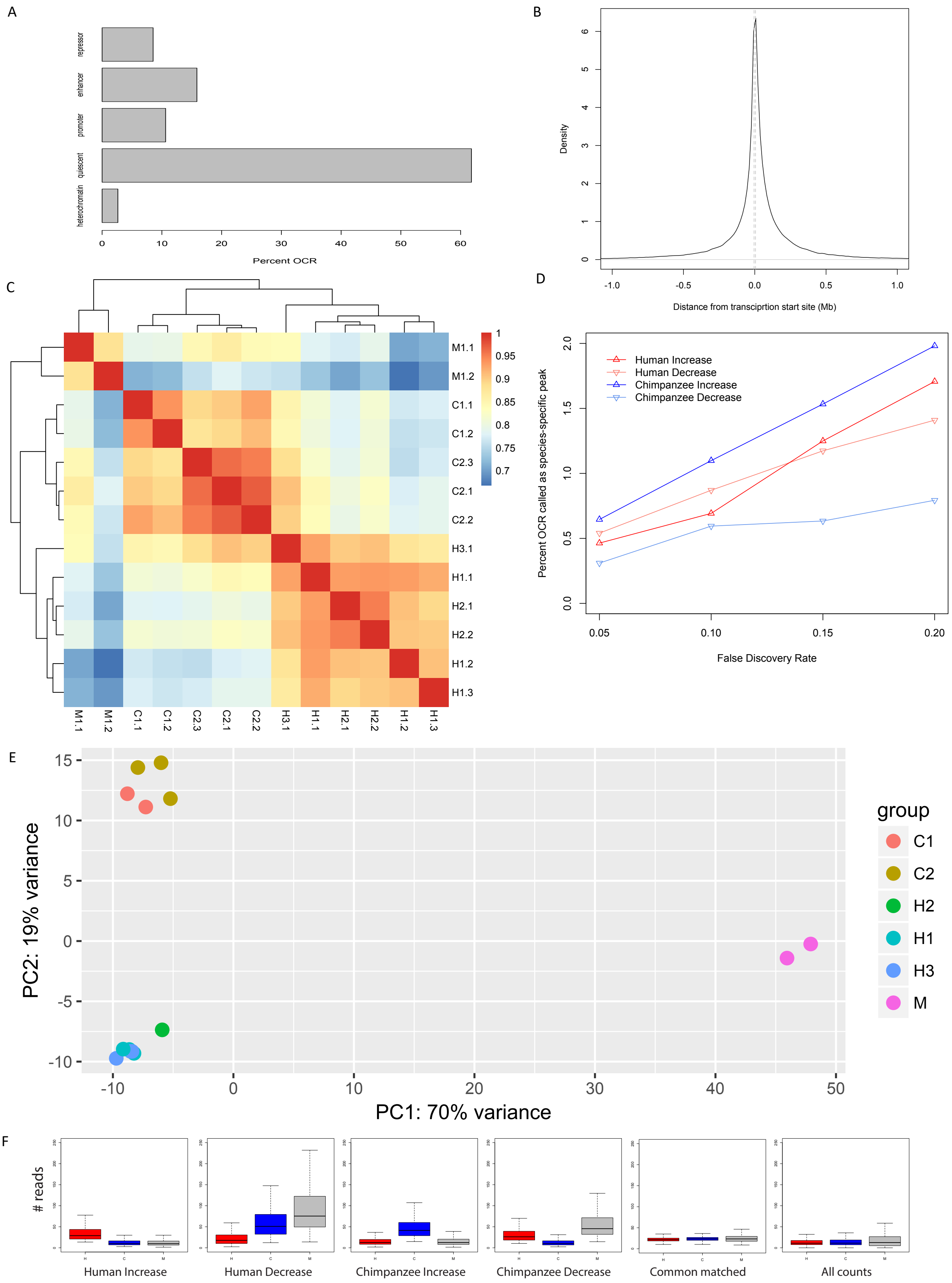
A
repressor
enhancer
promoter
quiescent
heterochromatin
0
10
20
30
40
50
60
Percent OCR
B
Density
0
1
2
3
4
5
6
-1.0
-0.5
0.0
0.5
1.0
Distance from transciprtion start site (Mb)
C
M1.1
M1.2
C1.1
C1.2
C2.3
C2.1
C2.2
H3.1
H1.1
H2.1
H2.2
H1.2
H1.3
1
0.95
0.9
0.85
0.8
0.75
0.7
D
Human Increase
Human Decrease
Chimpanzee Increase
Chimpanzee Decrease
Percent OCR called as species-specific peak
0.0
0.5
1.0
1.5
2.0
0.05
0.10
0.15
0.20
False Discovery Rate
E
PC2: 19% variance
15
10
5
0
-5
-10
0
10
20
30
40
50
PC1: 70% variance
group
C1
C2
H2
H1
H3
M
F
reads
Human Increase
Human Decrease
Chimpanzee Increase
Chimpanzee Decrease
Common matched
All counts

Supplemental Figure 1. Open Chromatin Regions (OCRs) in primate adipose. Percent OCRs annotated as functional genomic regions (A). Density plot of OCR distance from nearest transcription start site (B). Vertical gray lines are at -5 and 5 kb. Heatmap of all replicates (C). Biological replicates are H1 (Human 1104), H2 (Human 1442) , H3 (Human 602), C1 (Chimpanzee 4x327), C2 (Chimpanzee 4x519), M (Rhesus macaque) . Number of OCRs identified as species-specific changes based on increasing FDR (D). Technical replicates are denoted by .X where X is the number of the technical replicate. PCA of OCR height for technical replicates (E). Mapped read counts for all human (red), chimpanzee (blue) and macaque (gray) samples (F).

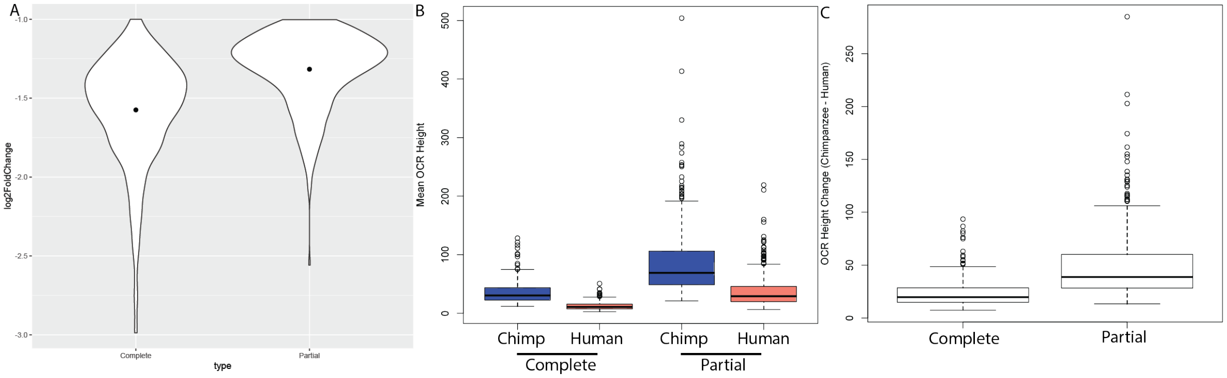

Supplemental Figure 2. Characteristics of Human Complete and Partial Losses (Human Decreased OCRs). Log2 fold change between human and chimpanzee orthologous OCRs (A). Peak height for chimpanzee (blue) and human (salmon) orthologous OCRs (B). Absolute intensity difference between chimpanzee and human orthologous OCRs (C).

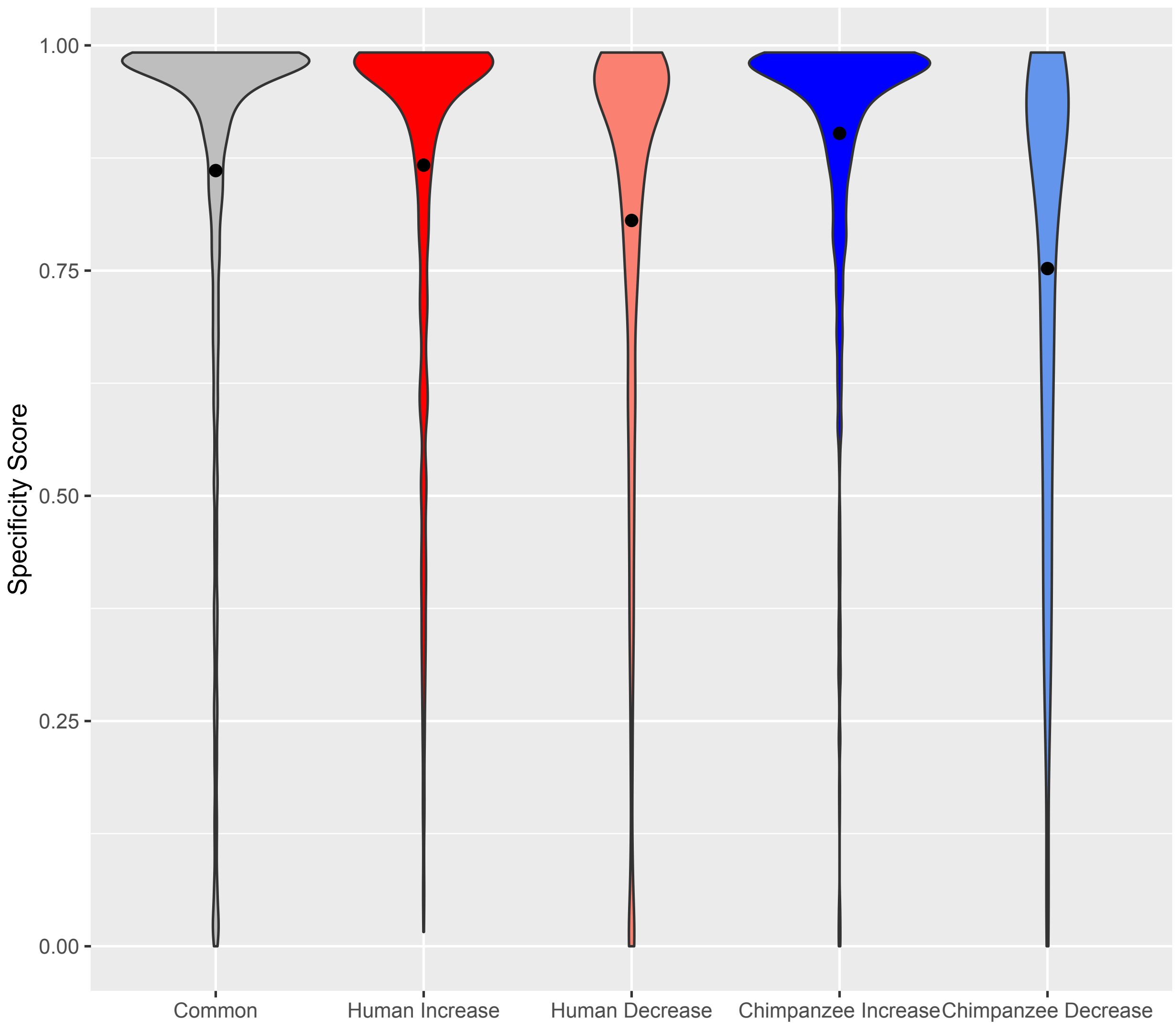

Supplemental Figure 3. Specificity scores for common and species-specific OCR groups. A score of 0 indicates low specificity (high pleiotropy) and that an OCR is open in all 125 cell and tissue types observed. A score of 1 indicate high specificity (low pleiotropy) and that an OCR is open in only adipose.

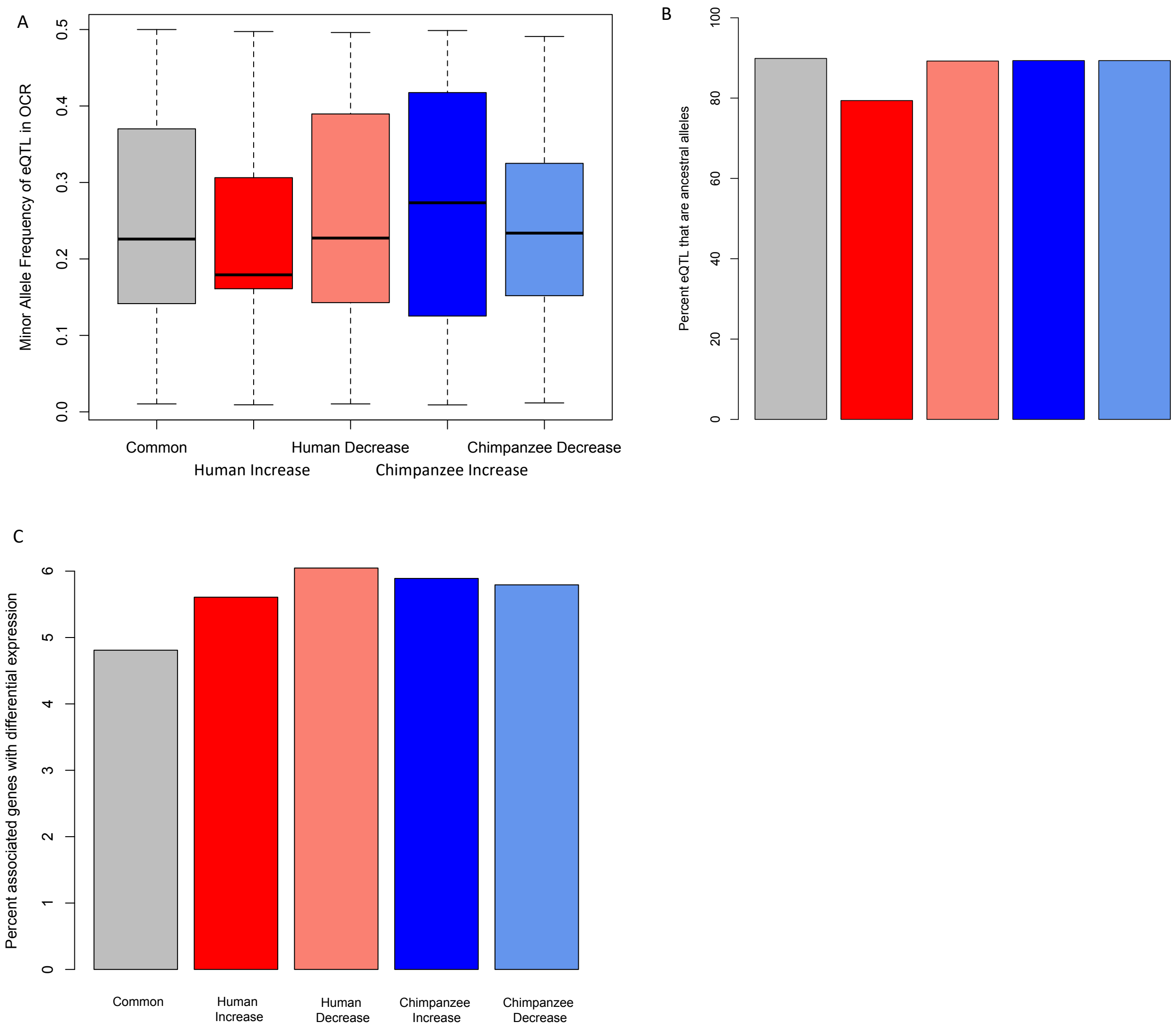

Supplemental Figure 4. OCRs and gene expression. Minor allele frequency of expression QTL (eQTL) in OCRs (A). Percent of OCRs containing ancestral allele (B). Percent associated genes with differential expression (C). OCRs were assigned to the closest gene.

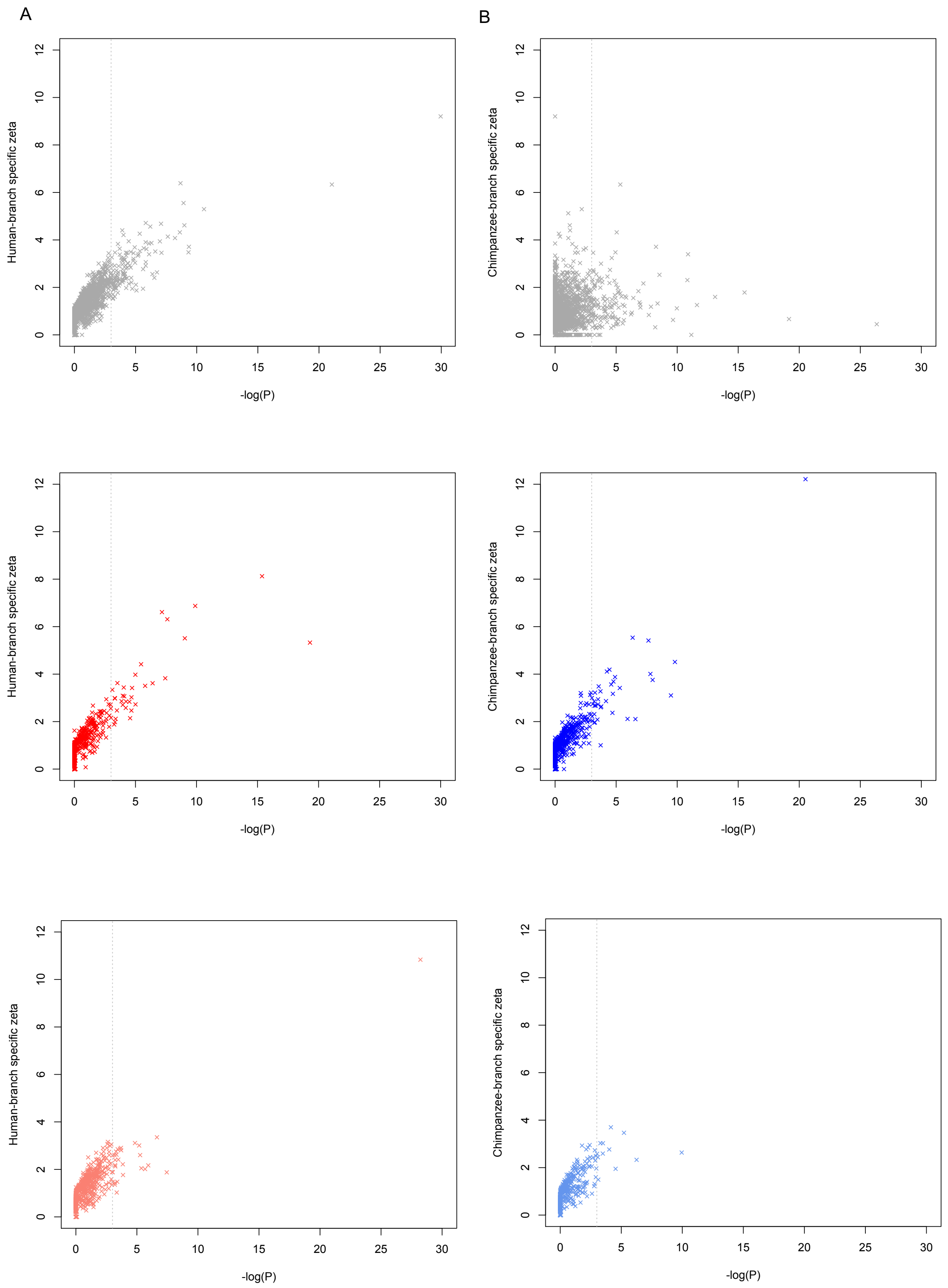
A
B
Human-branch specific zeta
Chimpanzee-branch specific zeta
-log(P)
0
2
4
6
8
10
12
0
5
10
15
20
25
30

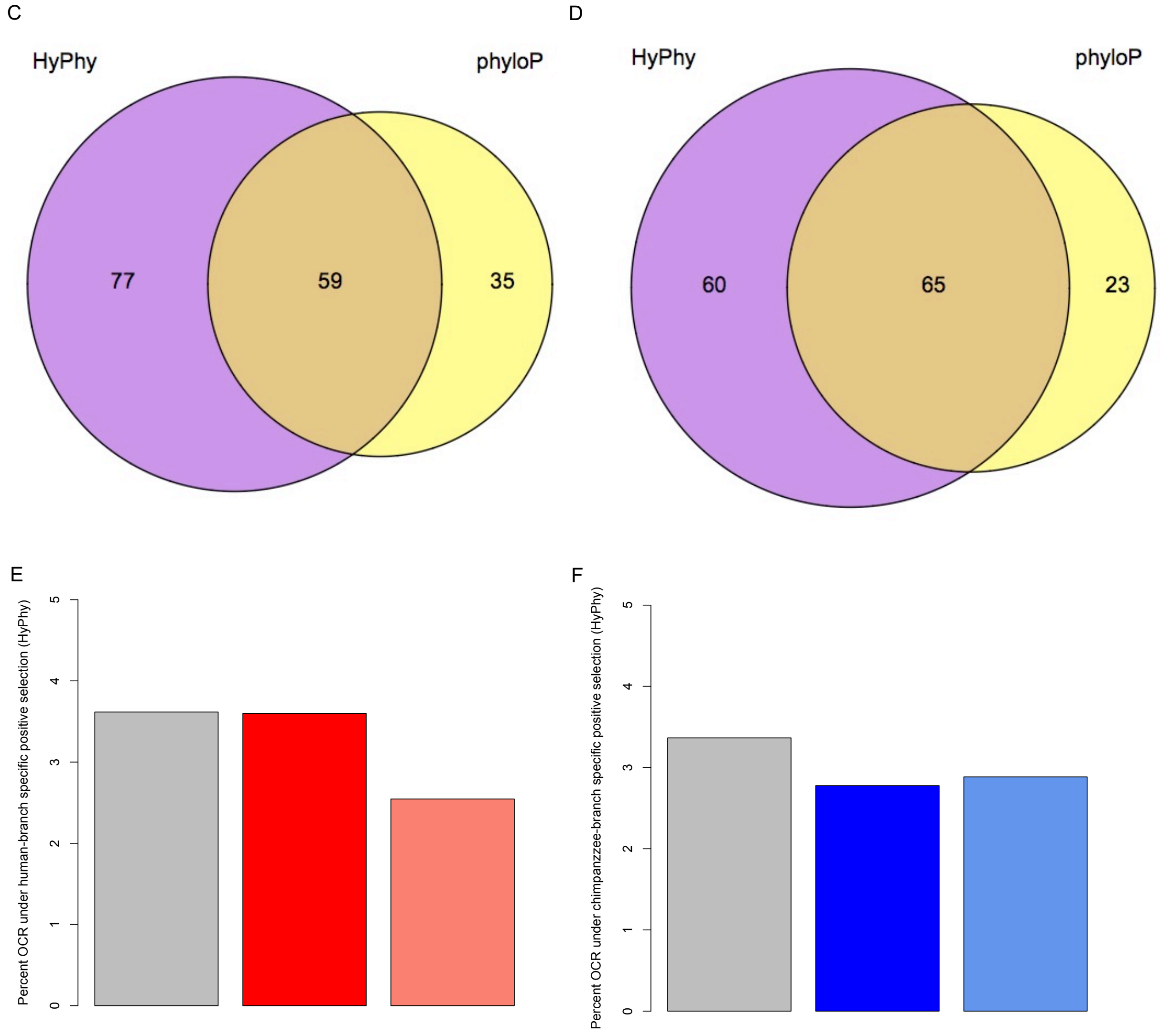

Supplemental Figure 5. Positive selection in OCRs detected by HyPhy. Human- (A) and Chimpanzee- (B) branch specific selection for each OCR belonging to common (top panel), high (middle panel), and low (bottom panel) groups. The vertical gray line depicts significance ($P < 0.05$). Percentages of OCRs under positive selection detected by HyPhy for Human- (E) and Chimpanzee- (F) branch specific positive selection.

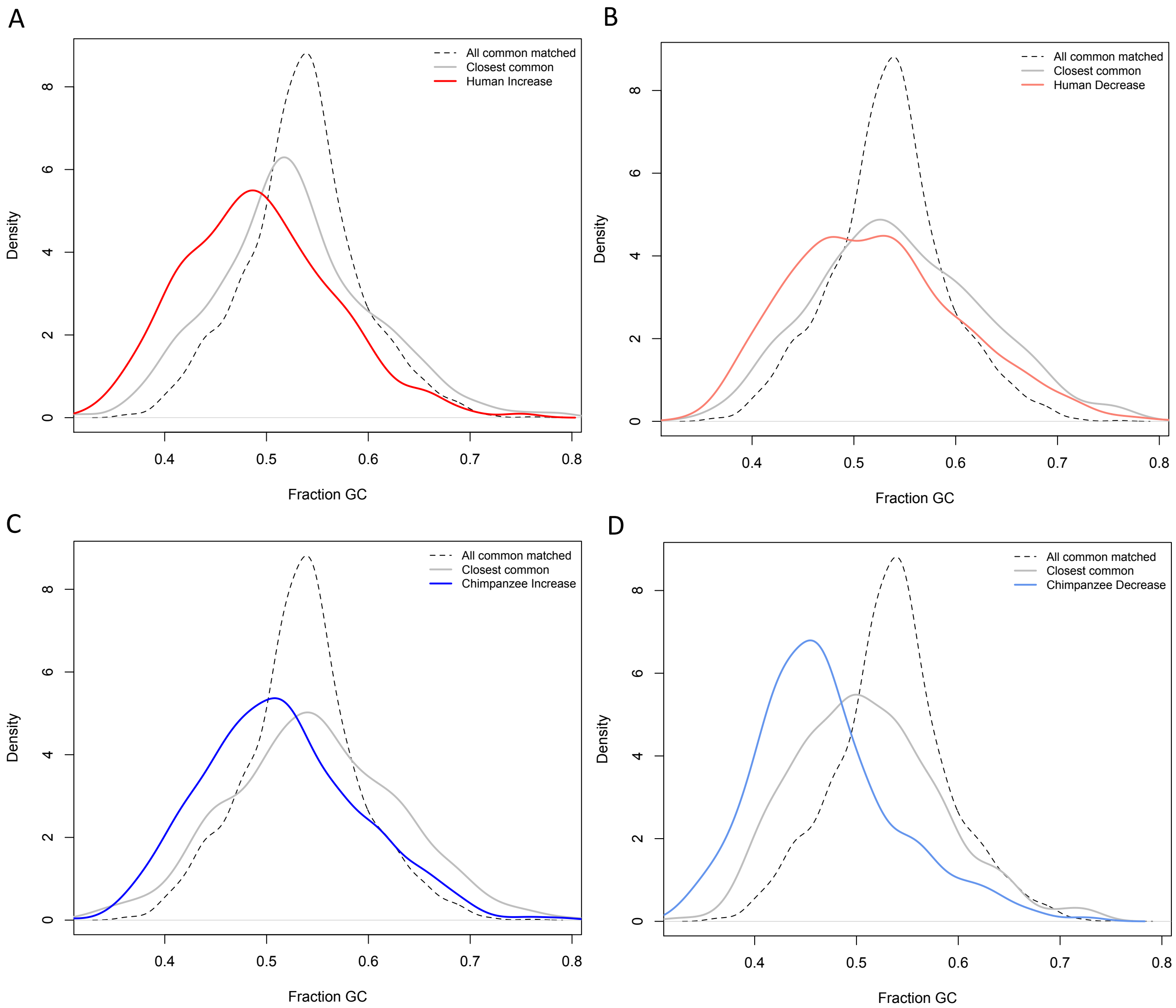

Supplemental Figure 6. GC content of species-specific OCRs compared to all common matched sites and the closest common site to a given OCR. Human Increase (A) Human Decrease (B) Chimpanzee Increase (C) Chimpanzee Decrease (D)

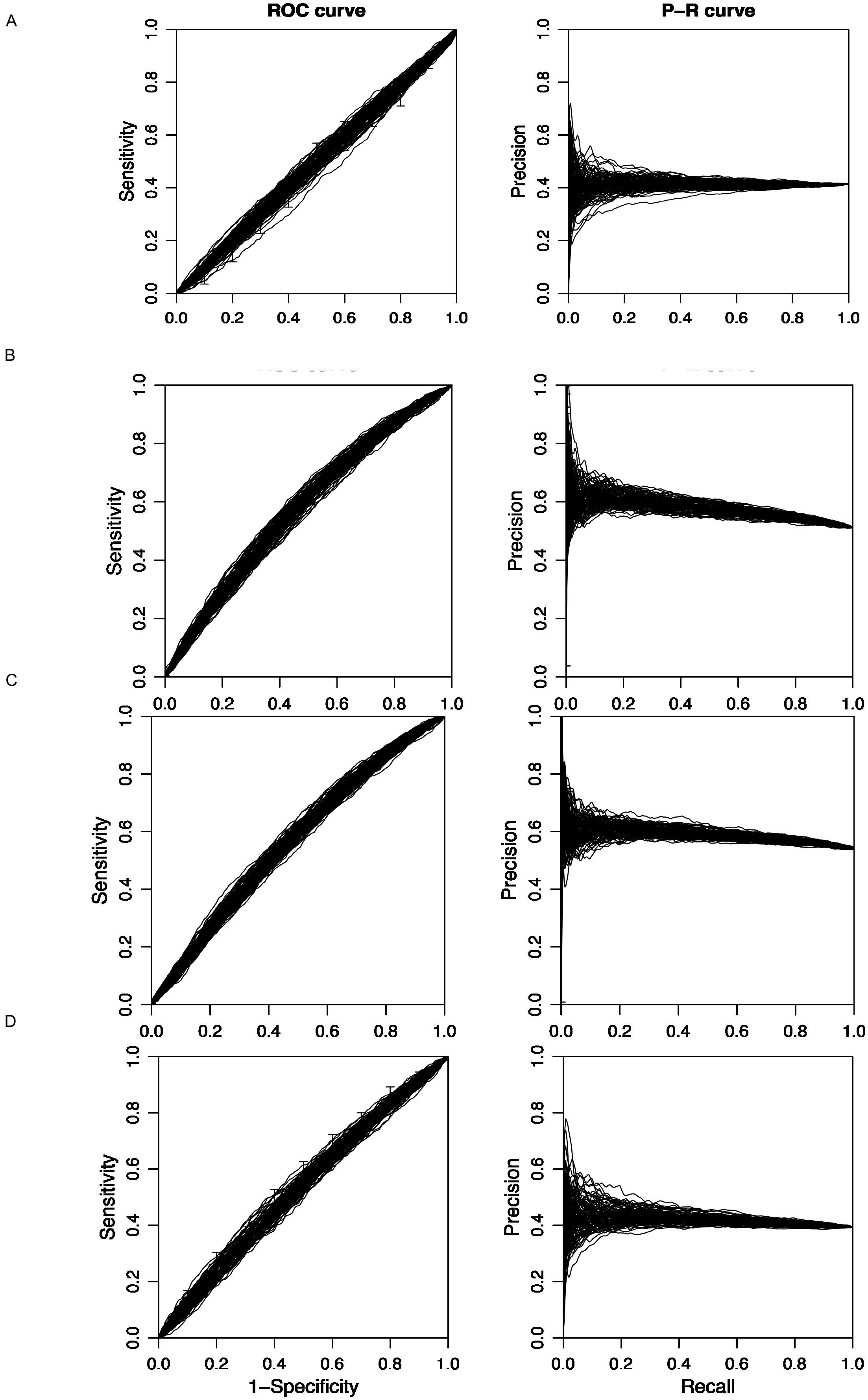

A
ROC curve
P-R curve
B
C
D
Sensitivity
Precision
1-Specificity
Recall
0.0
0.2
0.4
0.6
0.8
1.0

Supplemental Figure 7. Comparison of matched null groups to randomized genomic sequences. We used gkm-SVM to distinguish the matched null of human low (A), human high (B), chimpanzee low (C), and chimpanzee high (D) OCR groups from 1100 random sequences from the genome. We plotted the receiver/operating (left) and precision/recall (right) curves. Each curve is a separate set of 1100 sequences for a total of 100 sets of comparisons.

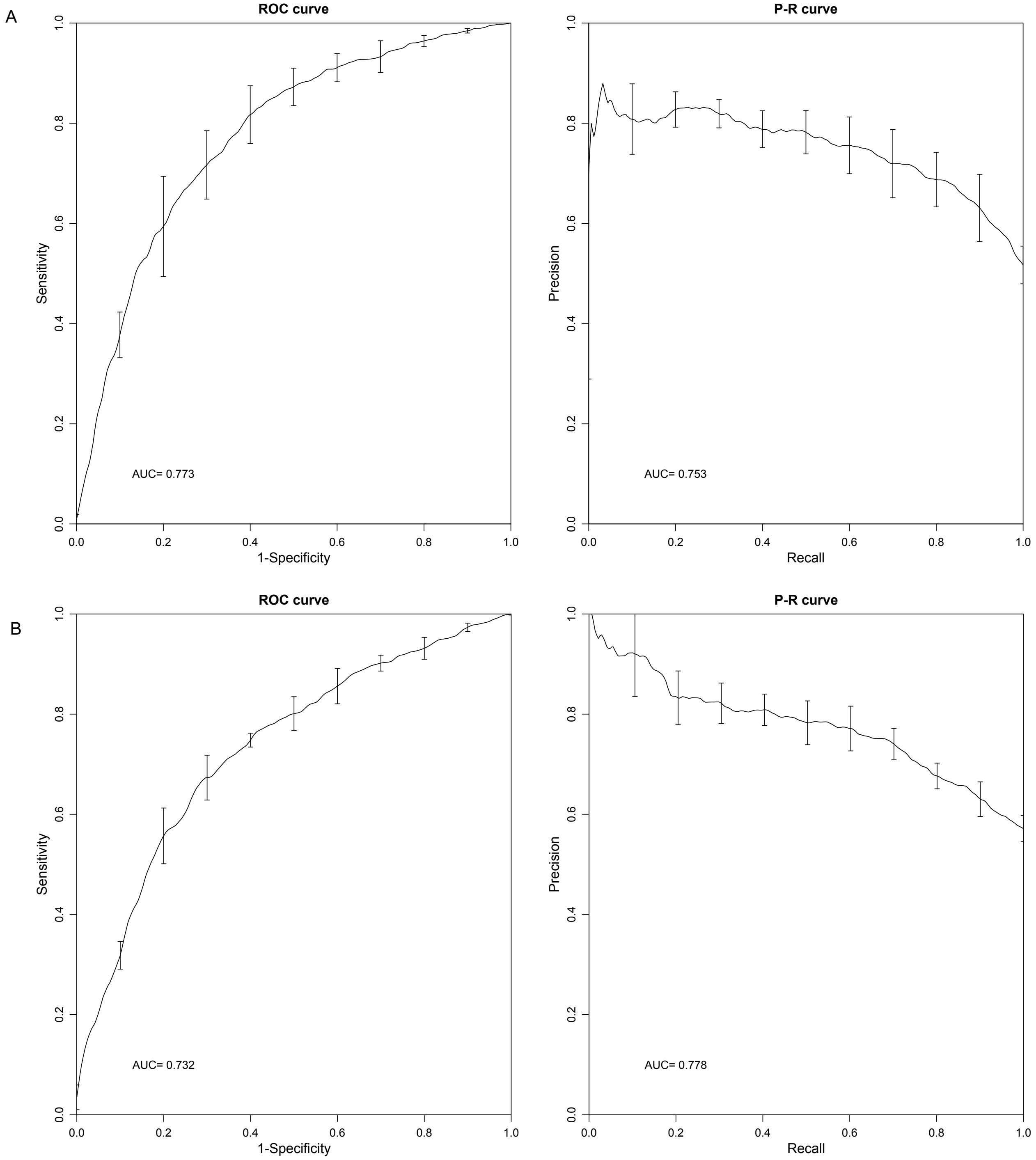
A
ROC curve
Sensitivity
1-Specificity
AUC= 0.773
P-R curve
Precision
Recall
AUC= 0.753
B
ROC curve
Sensitivity
1-Specificity
AUC= 0.732
P-R curve
Precision
Recall
AUC= 0.778
0.0
0.2
0.4
0.6
0.8
1.0

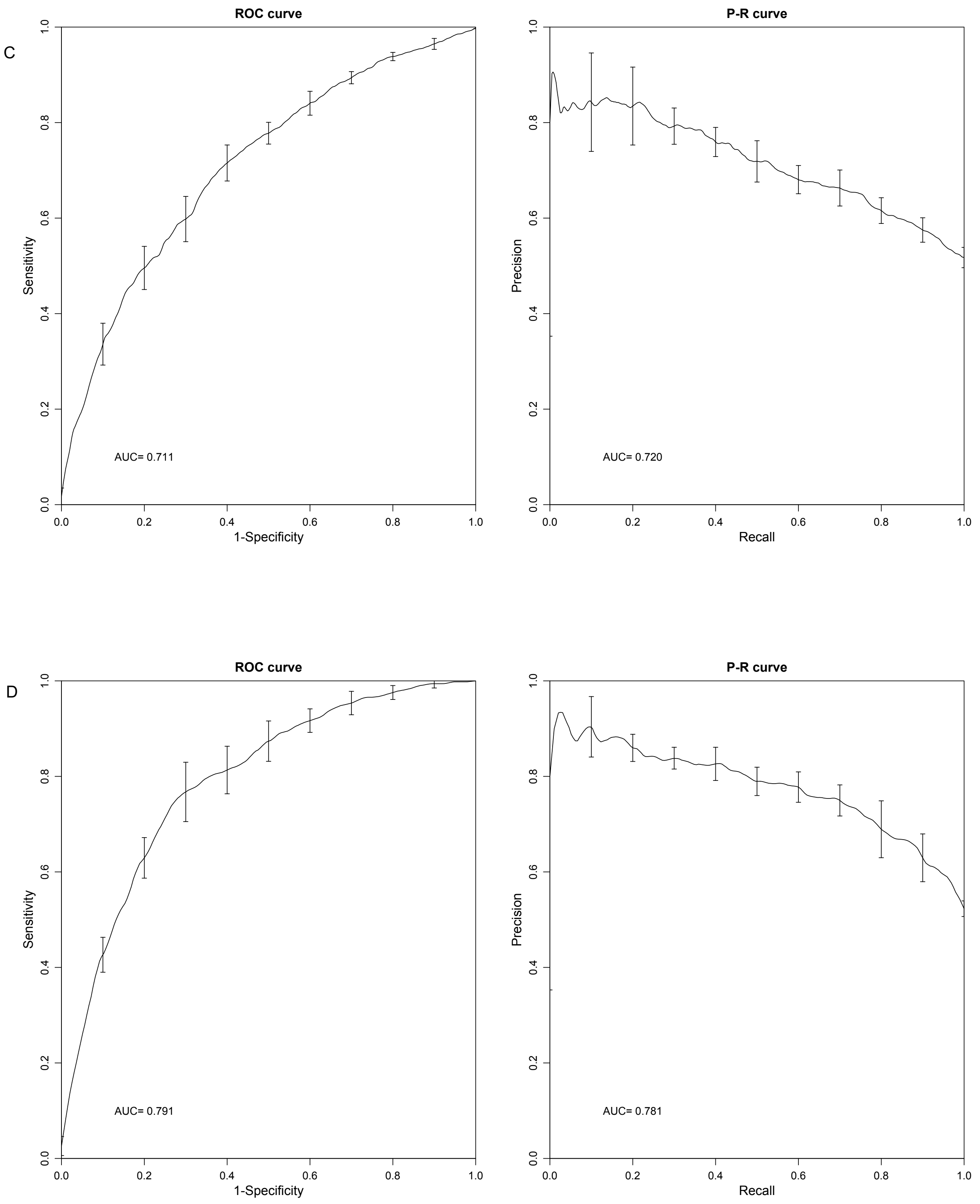

Supplemental Figure 8. Receiver operator characteristic (ROC) and precision recall (P-R) curves for species-specific OCR group compared to closet common OCRs. Human Increase (A) Human Decrease (B) Chimpanzee Increase (C) Chimpanzee Decrease (D).

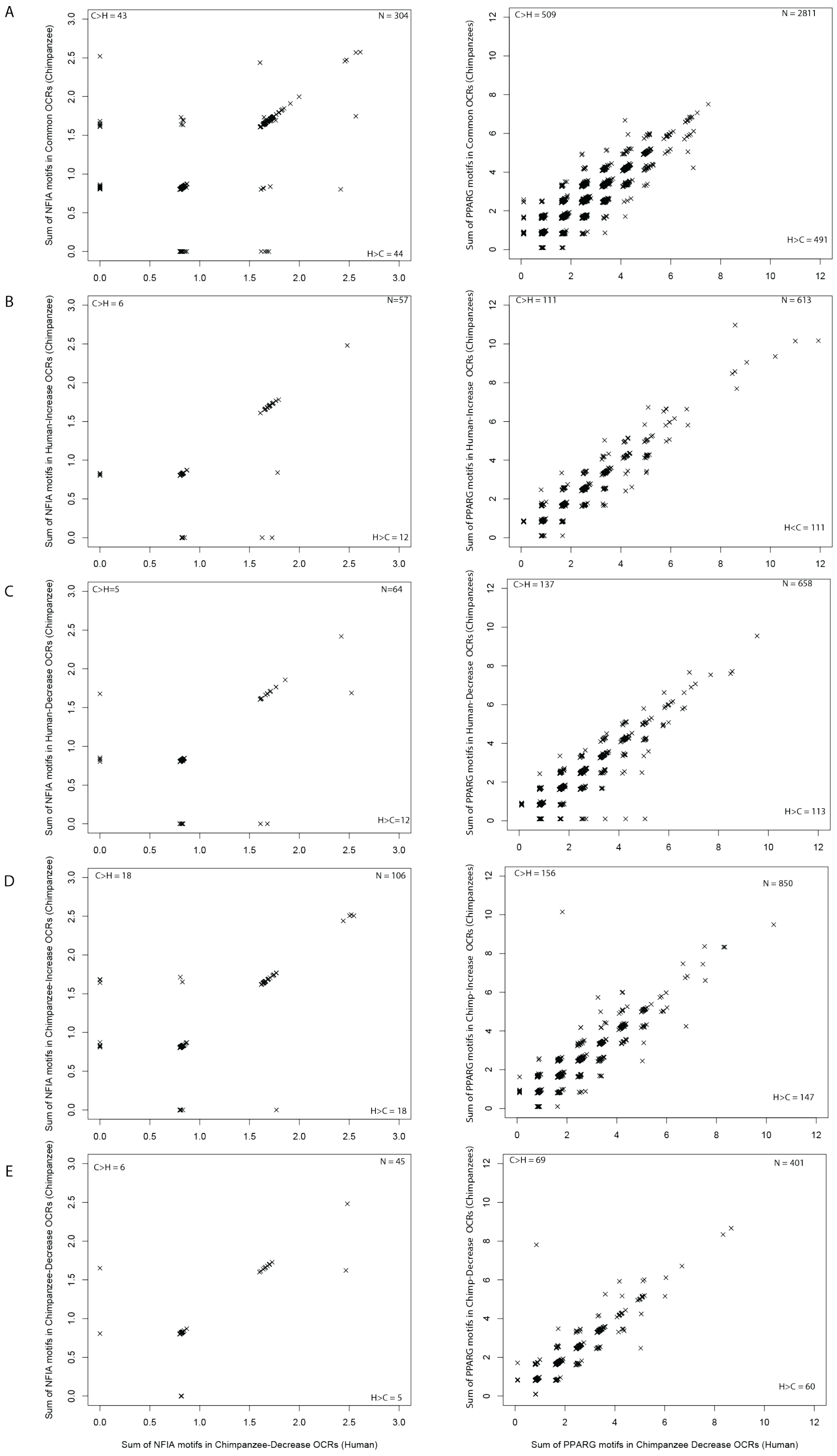
A
C>H = 43
N = 304
H>C = 44
Sum of NFIA motifs in Common OCRs (Chimpanzee)
C>H = 509
N = 2811
H>C = 491
Sum of PPARG motifs in Common OCRs (Chimpanzees)
B
C>H = 6
N=57
H>C = 12
Sum of NFIA motifs in Human-Increase OCRs (Chimpanzee)
C>H = 111
N = 613
H<C = 111
Sum of PPARG motifs in Human-Increase OCRs (Chimpanzees)
C
C>H=5
N=64
H>C=12
Sum of NFIA motifs in Human-Decrease OCRs (Chimpanzee)
C>H = 137
N = 658
H>C = 113
Sum of PPARG motifs in Human-Decrease OCRs (Chimpanzees)
D
C>H = 18
N = 106
H>C = 18
Sum of NFIA motifs in Chimpanzee-Increase OCRs (Chimpanzee)
C>H = 156
N = 850
H>C = 147
Sum of PPARG motifs in Chimp-Increase OCRs (Chimpanzees)
E
C>H = 6
N = 45
H>C = 5
Sum of NFIA motifs in Chimpanzee-Decrease OCRs (Chimpanzee)
C>H = 69
N = 401
H>C = 60
Sum of PPARG motifs in Chimp-Decrease OCRs (Chimpanzees)
Sum of NFIA motifs in Chimpanzee-Decrease OCRs (Human)
Sum of PPARG motifs in Chimpanzee Decrease OCRs (Human)

Supplemental Figure 9. Changes in orthologous motif sequences underlying OCRs. Sum of relative scores of NFIA (left panels) and PPARG (right panels) per OCR group. (A) Common (B) Human Increase (C) Human Decrease (D) Chimpanzee Increase (E) Chimpanzee Decrease. x-axis represents scores for the human orthologous sequence, and y-axis represents scores for the chimpanzee orthologous sequence. N is the number of OCRs containing the motif in question. "C > H" are the number of OCRs where the motif scores are stronger in the chimpanzee ortholog, and "H > C" are the number of OCRs where the motif scores are stronger in the human ortholog. Clusters are due to each motif score being scaled from 0 (no motif) to 1 (perfect motif). Absolute motif scores do not change results.
